# Clinical Manifestations and Disability After Acute Encephalitis Syndrome Among Pediatric Patients in Eastern Uttar Pradesh: A Retrospective Analysis

**DOI:** 10.7759/cureus.43777

**Published:** 2023-08-19

**Authors:** Shikha Gupta, Abhishek K Singh, Bhoopendra Sharma, Imran Ahmed Khan

**Affiliations:** 1 Physical Medicine and Rehabilitation, Baba Raghav Das Medical College, Gorakhpur, IND; 2 Pediatrics, Baba Raghav Das Medical College, Gorakhpur, IND; 3 Community and Family Medicine, Baba Raghav Das Medical College, Gorakhpur, IND

**Keywords:** rehabilitation, persistent disabilities, neuropsychiatric sequel, japanese encephalitis, immunization, follow-up, disability, acute encephalitis syndrome

## Abstract

Introduction

Acute encephalitis syndrome (AES) in pediatric patients can lead to a range of disabilities, affecting various aspects of their daily lives. The disease is caused by a diverse group of pathogens including viruses, bacteria, fungi, and protozoans. While significant progress has been made in combating the acute phase of the disease, the lingering effects on the physical, cognitive, and emotional well-being of survivors have yet to be comprehensively explored. The present retrospective study was conducted to investigate disabilities including neurological squeals and functional impairment challenges faced by AES survivors as they navigate life with disabilities.

Methods

We conducted a comprehensive retrospective analysis of medical records of pediatric patients diagnosed with AES and evaluated their follow-up visits at regular intervals during the study period. The Liverpool scoring system and clinical examinations were utilized to assess the presence and severity of disabilities in the patients.

Results

A total of 134 pediatric AES patients were included in the study; among them, 56% were males, and 44% were females. The mean age of the participants was 4.8 ± 3.1 years, and the mean number of days of hospitalization was 27.8 ± 30.8. Only 9.7% of the patients were found to be Japanese encephalitis (JE)-positive, and 87.5% of the participants were found to have disabilities in some or the other domain of the Liverpool Outcome Score (LOS). There were statistically significant correlations between the age of the patients and the LOS at follow-up. Post-recovery disabilities were more severe among patients who required a prolonged duration of hospitalization.

Conclusion

A considerable proportion of AES survivors are left with disabilities. Causes other than Japanese encephalitis are now more frequent in AES. The need for prolonged hospitalization is related to more severe disabilities. The early identification of disabilities through the Liverpool scoring system and clinical examination can aid in implementing appropriate intervention strategies.

## Introduction

Acute encephalitis syndrome (AES) refers to a group of clinically similar neurological manifestations of sudden-onset inflammation of the brain, presenting as high fever, altered mental status, seizures, coma, or even death. AES has long been recognized as a significant public health concern. AES affects both adults and children, but pediatric cases tend to have more severe outcomes due to their developing nervous systems [1]. Survivors of AES may experience a range of disabilities, leading to functional impairments in various aspects of daily life. The long-term outcomes and follow-up of pediatric AES patients with disabilities remain a critical area of investigation, given its implications for rehabilitation and patient care. The devastating impact of AES on the neurological system has often led to severe disabilities and long-term impairments, necessitating specialized care and support for affected children leading to considerable out-of-pocket expenses [2]. The disease is caused by a diverse group of pathogens including viruses, bacteria, fungi, and protozoans. Among virally associated encephalitis, Japanese encephalitis (JE) is an important cause of mortality and morbidity. After the first reported epidemic in Uttar Pradesh, there was a serious JE epidemic in 2005 [3]. Studies of outcomes among JE patients report widely varying results, with death in 4%-30% and long-term neurological impairment in 22%-94% [4-6]. The AES outbreak in Gorakhpur and nearby districts in recent years has brought to light the urgency of understanding the lasting consequences of this enigmatic syndrome, especially on young patients. AES surveillance and JE vaccination have contributed to the decline in cases of encephalitis due to JE. While significant progress has been made in combating the acute phase of the disease, the lingering effects on the physical, cognitive, and emotional well-being of survivors have yet to be comprehensively explored. The present retrospective study was conducted to investigate disabilities including neurological squeals and functional impairment among AES survivors as they navigate life with disabilities. The insights gleaned from this research endeavor aim to pave the way for more effective and tailored interventions, ultimately improving the quality of life for these young survivors.

## Materials and methods

Study design

The present retrospective, secondary data analysis was performed after obtaining ethical clearance from the Institutional Ethics Committee of Baba Raghav Das (BRD) Medical College (approval number: 19/IHEC/2023). Administrative approval for accessing the data of AES patients was obtained from the chief medical superintendent. As it was a secondary data analysis, informed consent was not applicable. The confidentiality of the data was maintained.

Study population and study period

Only those AES patients who were treated at the Department of Pediatrics of BRD Medical College and the Physical Medicine and Rehabilitation (PMR) Outpatient Department (OPD) and reported during the two-year period (from March 2017 to February 2019) for follow-up were included.

Inclusion criteria

The inclusion criteria included all discharged AES patients less than 15 years of age at the time of admission who attended the Physical Medicine and Rehabilitation OPD and the Department of Pediatrics of BRD Medical College, Gorakhpur, after obtaining consent from a legal guardian.

Exclusion criteria

Patients with incomplete data and those who were 15 years of age during the study period were excluded.

Study definition

A case of AES was defined as an acute onset of fever and, after an interval of one to a few days of prodrome, a change in mental status (including confusion, disorientation, delirium, or coma) and/or seizures (focal or generalized) [7]. Patients with febrile seizures (defined as a seizure in a child aged six months to six years whose only findings were fever and a single generalized convulsion of less than 15 minutes who recovers consciousness within 60 minutes of the seizure) were excluded [8].

Study tools

The Liverpool Outcome Score (LOS) was used for the follow-up of patients with AES along with some predefined clinical parameters [9]. The Liverpool Outcome Score has been developed for assessing disability in children after encephalitis caused by Japanese encephalitis virus (JEV) and has been validated in India. It assesses basic motor and self-care skills, as well as simple cognitive and behavioral functions, through 10 questions posed to the caregiver and five observations about the child’s performance of simple activities. The scores for each question ranged from 5, indicating full recovery, to 4, indicating minor sequelae with normal physical function, personality change, or receiving medication, to 3, indicating moderate sequelae and mildly affecting function, compatible with independent living, to 2, indicating severe sequelae, impairing function sufficient to make the patient dependent, to 1, indicating death. Because we excluded AES patients who died after discharge, no participant received a score of 1. The final outcome score (disability present or not) was the lowest score received for any of the 15 questions or activities. The scores for each domain may be analyzed separately, and the aggregate of all the individual scores might yield a result with a range of 33-75.

Data collection and statistical analysis

The data from registered cases of AES patients were collected from the central record room including follow-up consultations at three months, six months, and one year. The presence and severity of disabilities were documented based on the Liverpool scoring system and clinical evaluations at the time of follow-up visits. The data were filled out in the Excel spreadsheet (Microsoft® Corp., Redmond, WA), and after data cleaning, it was imported into the Statistical Package for Social Sciences (SPSS) version 21 (IBM SPSS Statistics, Armonk, NY) for further analysis. Descriptive analysis was done by applying appropriate statistical tests at the Department of Pediatrics and PMR in collaboration with the Department of Community Medicine of BRD Medical College, Gorakhpur. Categorical variables were expressed in absolute numbers and percentage scales and continuous variables as mean and standard deviation (SD). Correlation statistics were calculated between the age of the patients and days of hospitalization with the LOS. The means of the LOS at different time frames were compared according to JE status using an unpaired t-test. Statistical Package for Social Sciences (SPSS) version 21 was used for analysis, and a two-sided p-value of <0.05 was considered significant.

## Results

Four hundred seventy-six follow-up cases below 15 years of age were reported at PMR from March 2017 to February 2019 for follow-up after discharge. Two hundred fifty-five patients did not turn up for the next follow-up. Among the 221 patients, 87 had incomplete data and were thus excluded. Ultimately, 134 cases were included in the final analysis. Among them, 56% were males, and 44% were females. A maximum of 32.8% of the participants belonged to Gorakhpur district. The mean age of the patients was 4.8 ± 3.1 years (Table 1).

**Table 1:** Demographic characteristics of the patients (N=134)

Demographic characteristics		Frequency	Percentage
Gender	Male	75	56
	Female	59	44
Residence	Gorakhpur	44	32.8
	Kushinagar	21	15.7
	Deoria	17	12.7
	Bihar	15	11.2
	Maharajganj	11	8.2
	Siddharthnagar	11	8.2
	Sant Kabir Nagar	8	6
	Basti	3	2.2
	Gazipur	1	0.7
	Gonda	1	0.7
	Mau	1	0.7
	Balrampur	1	0.7
Age at the time of admission (year)	4.84 ± 3.1 (mean ± standard deviation)		

**Figure 1:**
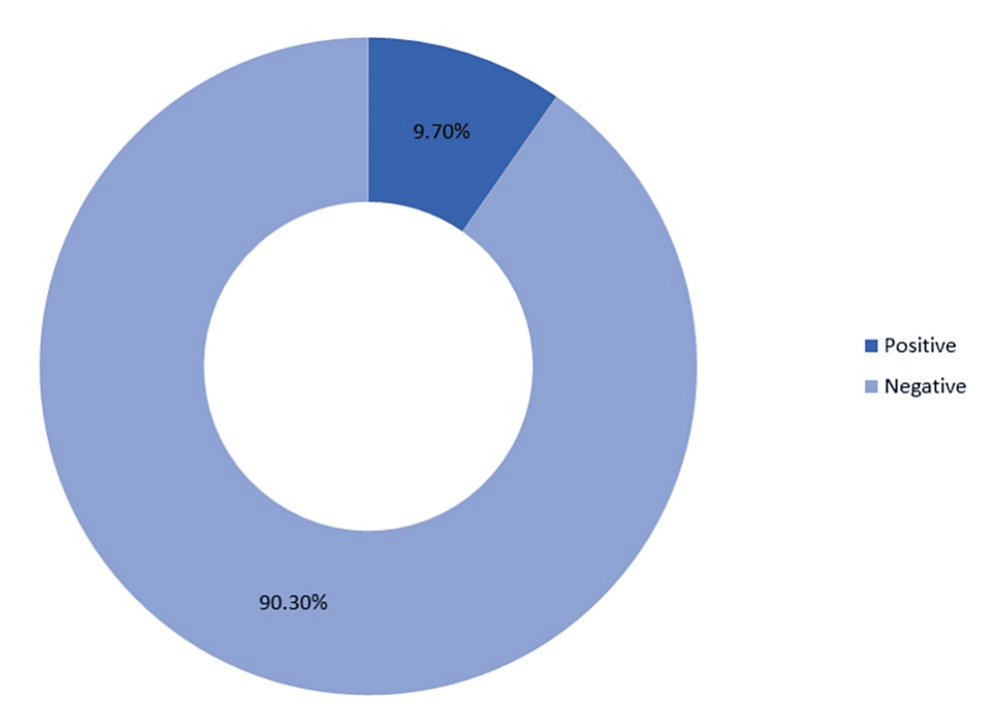
Japanese encephalitis status of the patients

Only 9.7% of the patients were found to be JE-positive (Figure 1).

**Figure 2:**
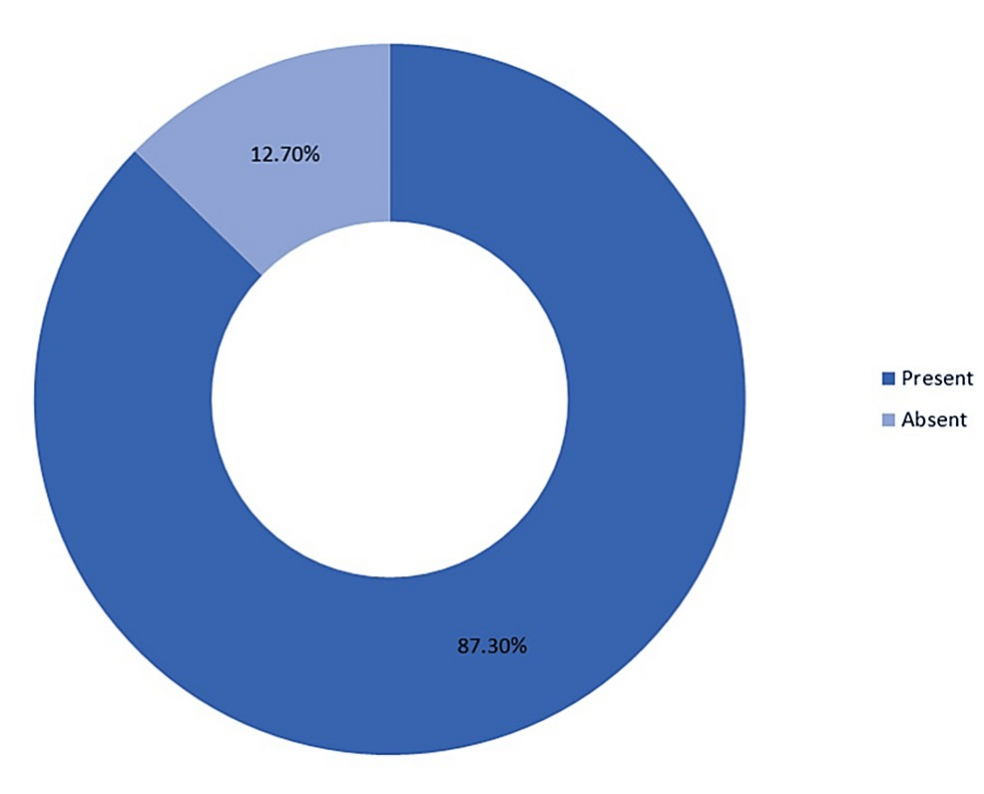
Disability among the participants

A majority of the patients (87.3%) were found to have disabilities in some or the other domain of the Liverpool Outcome Score (Figure 2).

The mean number of days of hospitalization was 27.8 ± 30.8. There is gradual improvement in the Liverpool Outcome Score over time (Table 2).

**Table 2:** Hospital stay and LOS of AES patients (N=134) AES, acute encephalitis syndrome; SD, standard deviation

Patient characteristics		Mean	SD	Median	Minimum	Maximum
Days of hospitalization		27.83	30.861	19.00	1	211
Liverpool Outcome Score (LOS)	At discharge	43.34	9.953	39.00	33	74
	At three months	50.51	12.553	47.00	33	74
	At six months	55.86	12.178	57.00	34	74
	At one year	61.07	10.699	64.00	34	74

Some predetermined clinical sequels were also assessed apart from the Liverpool Outcome Score (compiled in Table 3).

**Table 3:** Clinical sequel during follow-up (N=134) EPM, extrapyramidal movement; AED, antiepileptic drugs

Clinical sequel of the patients	Number of patients (percentage)			
	At discharge	At three months	At six months	At one year
Insomnia	47 (35.1)	38 (28.4)	10 (7.5)	5 (3.7)
Convulsion	27 (20.1)	36 (26.9)	39 (29.1)	34 (25.4)
Paresis	73 (54.5)	73 (54.5)	68 (50.7)	62 (46.3)
EPM	34 (25.4)	33 (24.6)	31 (32.1)	32 (23.9)
Excessive salivation	31 (23.1)	28 (20.9)	25 (18.7)	23 (17.2)
AED	81 (58.7)	81 (58.7)	81 (58.7)	60 (44.8)

There were statistically significant correlations between the age of patients and LOS at follow-up. Post-recovery disabilities were more severe among patients who required a prolonged duration of hospitalization (Table 4).

**Table 4:** Liverpool Outcome Score across the age of patients and the duration of hospitalization *Correlation is significant at the 0.05 level (two-tailed). **Correlation is significant at the 0.01 level (two-tailed)

Patient characteristics		Liverpool Outcome Score			
		At discharge	At three months	At six months	At one year
Age of patients in year	Correlation coefficient	0.189^*^	0.284^**^	0.240^**^	0.261^**^
	Significance (two-tailed)	0.028	0.001	0.005	0.002
Days of hospitalization	Correlation coefficient	-0.406**	-0.497**	-0.473**	-0.418**
	Significance (two-tailed)	0.000	0.000	0.000	0.000

There were statistically significant differences between the means of JE-positive and JE-negative patients at the time of discharge and follow-up at three months, whereas there were no statistically significant differences at six months and one year (Table 5).

**Table 5:** Follow-up Liverpool Outcome Score according to JE status of the patients JE, Japanese encephalitis; SD, standard deviation

Liverpool Outcome Score	Japanese encephalitis status	Number	Mean	SD	P-value (t-test)
At discharge	Positive	13	38.92	3.546	0.001
	Negative	121	43.82	10.305	
At three months	Positive	13	45.15	8.745	0.040
	Negative	121	51.09	12.789	
At six months	Positive	13	52.69	10.633	0.283
	Negative	121	56.20	12.323	
At one year	Positive	13	60.38	9.251	0.785
	Negative	121	61.15	10.874	

## Discussion

Seasonal outbreaks of acute encephalitis syndrome (AES) have been occurring regularly in some areas of eastern Uttar Pradesh, India. These outbreaks usually occur during the monsoon and post-monsoon periods, predominantly affecting children in rural areas, and are associated with a high fatality rate (15%-25%) [10]. There occurs considerable disability among the survivors. Studies conducted among survivors of JE indicated a moderate to severe degree of disability in 27%-50% of patients. The common sequelae included seizures, urinary incontinence, abnormal behavior, needing help with dressing or inability to be left alone without coming to harm, and the deterioration of school or work performance [11,12]. We collected and analyzed data from hospital records for patients who attended for follow-up after discharge and fit our inclusion criteria.

The proportion of males (56%) was more in comparison to females (44%), and the age of affected patients was 4.8 ± 3.1 years in our study. Khinchi et al. also found male preponderance in their study [13]. Shrivastava et al., in their review, found that the majority of AES patients were from eastern Uttar Pradesh and had non-JE causes [14]. Similar geographical distribution of AES cases was found in this study, and JE was positive in only 9.75% of AES cases. An earlier study conducted in Gorakhpur from 2008 to 2010 reported severe disability in 12.9% and moderate disability in 32.7% [15]. We found no disability among 12.7% of the patients at the time of discharge. This finding is in line with Srivastava et al., who found full recovery in 12% of their AES patients [15]. Similarly, Maha et al. also found a high proportion of squeals among AES survivors [16]. These findings emphasize the need to stress preventive measures because AES survivors are left with lifelong disabilities. Those unfortunate patients with post-AES disabilities need special attention to live normal lives.

Our study reflected a slight improvement in LOS during follow-up among survivors. Our finding is in line with Mayxay et al., who also observed improvement in LOS between discharge and the last follow-up [17]. Sarkari et al. also found a reduction in disability on long-term follow-up of AES patients. Return to near-normal working was observed after prolonged follow-up, reducing the burden on family and society in the majority of patients [18].

Paresis was the most common clinical sequel in our patients during follow-up. We found that 20.1% of children had seizures, which increased to 26.9% at three months and 29.1% at six months of follow-up and decreased to 25.4% at one-year follow-up. Ooi et al. also concluded that children with Japanese encephalitis may get better or get worse months after the initial attack [5]. In our study, there were statistically significant differences in disabilities among AES follow-up patients among JE and non-JE causes. As time went on, these discrepancies became smaller. Some other studies also observed differences in the pattern of disabilities among JE and other causes of AES [14,19].

Strengths and limitations

By examining the long-term outcomes and follow-up care of pediatric AES patients with disabilities, we hope to contribute meaningfully to the ongoing dialogue on AES, empower affected families with knowledge, and inform healthcare professionals with valuable data to shape future treatment protocols. As we delve into the details of this retrospective study, it is our earnest hope that this research will inspire further investigations and foster a compassionate and comprehensive approach to care for pediatric AES survivors.

Our study had certain limitations. We have collected data only from those patients who reported to our institutions. Some patients with disability might be missed because they did not come for follow-up, migrated, or died due to any unrelated cause.

Recommendations

It is necessary to screen AES patients to early identify disabilities including impairments in cognition and behavior during their follow-up visits post discharge and strengthen the cognitive rehabilitation service. These patients can be referred to the specialized public disability rehabilitation centers for appropriate rehabilitative care so that further deterioration can be prevented, which would also reduce the out-of-pocket expenditure incurred on these families. JE vaccination should be encouraged.

## Conclusions

The present retrospective analysis indicates that disability was frequent among survivors of AES. Males were found to be more affected by AES than females, and the majority of AES patients were negative for Japanese encephalitis. The level of disability was more severe among younger children and in those with prolonged hospital stays. Disability among AES survivors was more among JE patients than other AES causes, and this difference was statistically significant at discharge and three-month follow-up.
